# Inhibitory Effects of Sulfonamide Derivatives on the *β*-Carbonic Anhydrase (MpaCA) from *Malassezia pachydermatis*, a Commensal, Pathogenic Fungus Present in Domestic Animals

**DOI:** 10.3390/ijms222212601

**Published:** 2021-11-22

**Authors:** Viviana De Luca, Andrea Angeli, Valeria Mazzone, Claudia Adelfio, Fabrizio Carta, Silvia Selleri, Vincenzo Carginale, Andrea Scaloni, Claudiu T. Supuran, Clemente Capasso

**Affiliations:** 1 Institute of Biosciences and Bioresources, CNR, Via Pietro Castellino 111, 80131 Napoli, Italy; viviana.deluca@ibbr.cnr.it (V.D.L.); valeria.mazzone@ibbr.cnr.it (V.M.); cla.delfio@gmail.com (C.A.); vincenzo.carginale@ibbr.cnr.it (V.C.); 2 Proteomics, Metabolomics & Mass Spectrometry Laboratory, Institute for the Animal Production System in the Mediterranean Environment, CNR, P.le Enrico Fermi 1, 80055 Portici (Napoli), Italy; andrea.scaloni@ispaam.cnr.it; 3 Section of Pharmaceutical and Nutraceutical Sciences, Department of Neurofarba, University of Florence, Via U. Schiff 6, 50019 Sesto Fiorentino (Florence), Italy; andrea.angeli@unifi.it (A.A.); fabrizio.carta@unifi.it (F.C.); silvia.selleri@unifi.it (S.S.)

**Keywords:** carbonic anhydrase, *Malassezia pachydermatis*, CO_2_ sensing, sulfonamide inhibitors, anti-dandruff, antifungals

## Abstract

Fungi are exposed to various environmental variables during their life cycle, including changes in CO_2_ concentration. CO_2_ has the potential to act as an activator of several cell signaling pathways. In fungi, the sensing of CO_2_ triggers cell differentiation and the biosynthesis of proteins involved in the metabolism and pathogenicity of these microorganisms. The molecular machineries involved in CO_2_ sensing constitute a promising target for the development of antifungals. Carbonic anhydrases (CAs, EC 4.2.1.1) are crucial enzymes in the CO_2_ sensing systems of fungi, because they catalyze the reversible hydration of CO_2_ to proton and HCO_3_^-^. Bicarbonate in turn boots a cascade of reactions triggering fungal pathogenicity and metabolism. Accordingly, CAs affect microorganism proliferation and may represent a potential therapeutic target against fungal infection. Here, the inhibition of the unique *β*-CA (MpaCA) encoded in the genome of *Malassezia pachydermatis*, a fungus with substantial relevance in veterinary and medical sciences, was investigated using a series of conventional CA inhibitors (CAIs), namely aromatic and heterocyclic sulfonamides. This study aimed to describe novel candidates that can kill this harmful fungus by inhibiting their CA, and thus lead to effective anti-dandruff and anti-seborrheic dermatitis agents. In this context, current antifungal compounds, such as the azoles and their derivatives, have been demonstrated to induce the selection of resistant fungal strains and lose therapeutic efficacy, which might be restored by the concomitant use of alternative compounds, such as the fungal CA inhibitors.

## 1. Introduction

Carbon dioxide (CO_2_) is ubiquitously generated and released into the atmosphere through cellular respiration and oxidative metabolism [1]. This gas byproduct is typically transported out of cells via passive diffusion. This transport may be aided by CO_2_ channels, which are regulated in a CO_2_-dependent way [2,3]. Rather than a waste product, CO_2_ has also the potential to act as a physiological stimulant for a variety of cellular signaling pathways that promote microorganism virulence and pathogenicity [1,3,4]. For example, Bacteria assist their colonization and infection at CO_2_ concentration levels comparable to those found in the host, since bacterial CO_2_ sensing mechanisms allow them to adapt and survive in such environments [1]. During their life cycle, fungi are exposed to various environmental variables, including fluctuations in CO_2_ levels [1]. Dedicated CO_2_ sensing machineries allow fungi to sense the amount of CO_2_ present in mammalian tissues (about 5%), compared to the atmospheric levels (about 0.03%), which ultimately stimulates the fungal pathogenicity in the host [1,4]. 

In microorganisms, proteins involved in sensing CO_2_ have been suggested as attractive targets of pharmaceuticals since they modulate cell differentiation and the further production of molecules essential for the pathogen [4,5]. In this context, it has been demonstrated that carbonic anhydrases (CAs, EC 4.2.1.1), catalyzing the reversible hydration of CO_2_ to HCO_3_^−^ and H^+^ (CO_2_ + H_2_O ⇋ HCO_3_^−^ + H^+^) [6,7,8,9,10,11,12], are crucial enzymes in fungal CO_2_ sensing since they produce bicarbonate, which is a promoter (through an adenylate cyclase (AC) intermediate enzyme) of fungal meiosis and sporulation [13]. Indeed, HCO_3_^−^ produced in a CA-dependent manner activates AC and cyclic adenosine monophosphate (cAMP) production, which stimulates the development of filamentous structures (hyphae) needed for fungal virulence, adhesion, and the production of hydrolases, thus triggering cell death in the colonized host [4,5,14,15]. Up to date, eight CA gene families or classes have been identified and designated with Greek letters (α, β, γ, δ, ζ, η, θ, ι) [6,7,8,9,10]. In the fungal kingdom, the typical class is represented by *β*-CAs, which generally occur with at least one isoform. Conversely, α-CAs are rarely found in fungi [5,16,17,18]. The catalytic action of fungal CAs triggers a cascade process, which allows the microorganism to adapt into the host, thrive therein, and contribute to its pathogenicity [4,14,15,18,19]. It is readily apparent that CAs can affect fungal growth and thus may represent a potential novel therapeutic target in fungal infections. This is corroborated by the studies of Supuran’s group, who demonstrated that typical CA inhibitors (CAIs), namely primary sulfonamides, inhibit the growth of *Malassezia globosa* when CO_2_ availability is limited (i.e., fungus-infected skin surface) [20]. In this context, we have focalized our interest on another such fungus, *M. pachydermatis*, which has a significant relevance in veterinary and medical sciences, as it has been associated with otitis externa and seborrheic dermatitis in dogs, cats, and wild animals, as well as with fungemia in hospitalized and immunocompromised patients [21,22,23,24]. When the skin microenvironment or the host’s defenses are compromised, this opportunistic commensal has the potential to become a disease-causing pathogen [21,22]. In this context, we determined that the genome of the *M. pachydermatis* contains a single gene encoding a *β*-CA (acronym *MpaCA*) that is closely related to *β*-CAs previously identified by our groups in two other Malassezia species, namely *M. globosa* and *M. restricta*, which are responsible for dandruff and seborrheic dermatitis [20,25,26,27,28,29,30,31,32,33,34]. 

Here, we have further investigated MpaCA, focusing on its inhibition profile with respect to a series of aromatic or heterocyclic sulfonamides, which are widely used as building blocks for obtaining potent and selective pharmacological agents. Besides, inhibition data on MpaCA have been compared with those of ortholog *β*-CA enzymes from *M. globosa* and *M. restricta*, namely MgCA and MreCA, respectively. Overall, this study tentatively proposes novel potential anti-dandruff and anti-seborrheic dermatitis compounds able to eradicate harmful fungi through the inhibition of CAs. This is potentially relevant since clinically used antifungal drugs, such as azoles and their derivatives, as result of their widespread diffusion, have determined the selection of resistant fungal strains.

## 2. Results and Discussion

### 2.1. Biochemical Characterization of MpaCA

**Figure 1:**
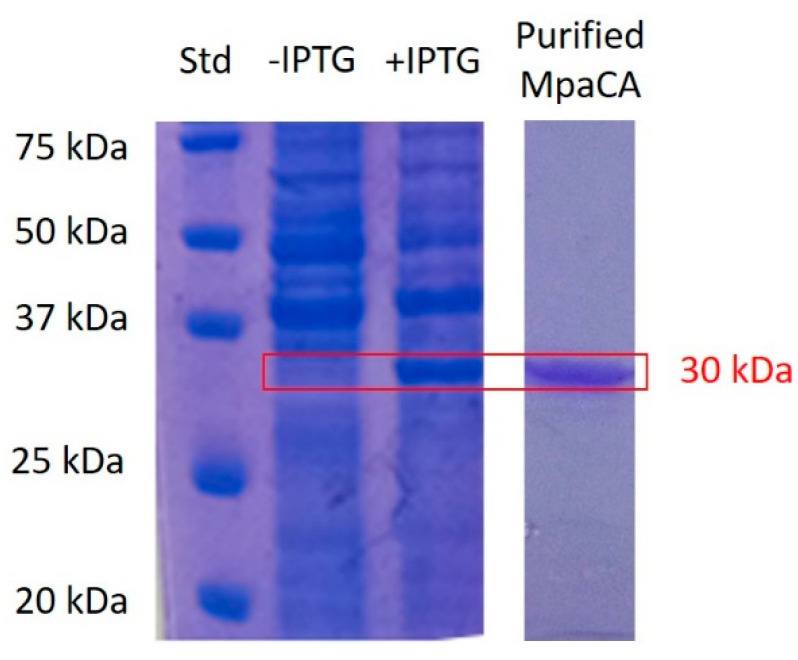
SDS-PAGE showing results regarding MpaCA heterologous expression in bacterial cells and further purification from corresponding extracts. A total of 1 mM IPTG was used to induce MpaCA biosynthesis. Overexpressed MpaCA is visible in the lane indicated with “+IPTG”, migrating with an apparent molecular mass of about 30 kDa. The enzyme is not present in lane “-IPTG”, which shows the bacterial lysate supernatant before isopropyl-β-D-thiogalactopyranoside induction. A bacterial extract containing soluble MpaCA was resolved on a HisTrap FF column to yield a pure, homogeneous preparation of the fungal enzyme (Lane “Purified MpaCA”). The red box represents MpaCA migrating with an experimental molecular mass of 30 kDa.

Recombinant *M. pachydermatis* CA (MpaCA) was overexpressed in *E. coli* as a fusion protein with a non-natural tail containing six histidines at its molecular N-terminus, and purified by affinity chromatography. The purified enzyme was then subjected to SDS-PAGE and protonography to certify the corresponding molecular mass and ability to elicit a hydratase activity. As shown in Figure 1, SDS-PAGE demonstrated that recombinant MpCA was recovered in the soluble fraction of the bacterial extract in response (after 3 h) to isopropyl-β-D-thiogalactopyranoside (IPTG) induction. Recombinant MpCA showed an experimental molecular mass of about 30 kDa, in agreement with the expected theoretical one (31 kDa). 

**Figure 2:**
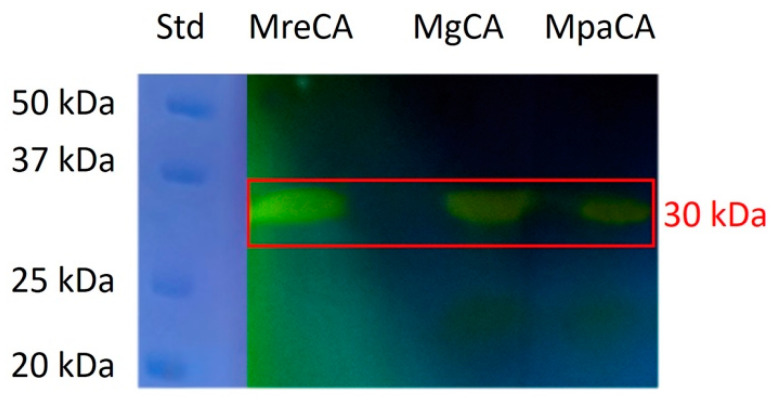
Protonographic analysis of MreCA, MgCA, and MpaCA. The CO_2_ hydratase activity was directly evaluated on the polyacrylamide gel through the development of yellow bands due to pH value variations (acidic pH) resulting from the conversion of CO_2_ to bicarbonate and protons (H^+^). Legend: Lane Std, molecular markers; Lane MreCA, purified MreCA; Lane MgCA, purified MgCA; Lane MpaCA, purified MpaCA. The red box shows the enzyme activity of the three fungal enzymes, which migrated with an apparent molecular mass of about 30 kDa.

Protonographic analysis (Figure 2) was used to determine whether purified recombinant MpaCA could catalyze the CO_2_ hydration reaction (Figure 1 and Figure 2). Two homologous CAs, namely MgCA and MreCA, which are encoded from the genome of *M. globosa* and *M. restricta*, respectively, were used as positive controls. As expected, the protonogram in all cases exhibited yellow bands migrating at a molecular mass of about 30 kDa (Figure 2), thus demonstrating a CO_2_ hydratase activity for all fungal CAs, including MpaCA. 

With the aid of the stopped-flow technique, we further demonstrated that MpaCA exhibits an appreciable CO_2_ hydrase activity, with a k_cat_ value of 3.8 × 10^5^ s^−1^ and k_cat_/K_M_ value of 9.7 × 10^6^ M^−1^ s^−^. MgCA and MreCA showed a catalytic activity very similar to that of MpaCA. In particular, MreCA showed a k_cat_ value = 1.06 × 10^6^ s^−1^ and k_cat_/K_M_ value = 1.07 x 10^8^ M^−1^s^−1^ [25], while MgCA exhibited a k_cat_ value of 9.2 × 10^5^ s^−1^ and k_cat_/K_M_ value of 8.3 × 10^7^ M^−1^ s^−1^ [27,28,29,30,31,32,33]. 

### 2.2. Inhibition Profile of MpaCA with Sulfonamides

**Figure 3:**
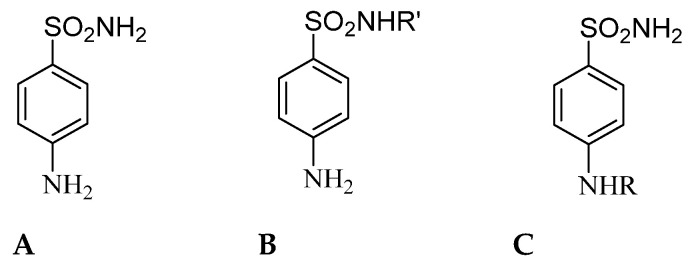
Sulfanilamide (Panel **A**) led to the discovery of the sulfadrugs (Panel **B**) and benzenesulfonamide CAIs of type (Panel **C**).

Sulfonamide compounds represent a significant class of synthetic bacteriostatic antibiotics still used today to treat infections caused by bacteria and other microorganisms [35,36,37]. They are also known as sulfa drugs. These compounds are derived from sulfanilamide (compound A in Figure 3) and include synthetic derivatives with the general chemical structure B [38]. Worth mentioning is the fact that often the term sulfonamide is imprecisely referred to antibiotics bearing a sulfonamide moiety, and not all sulfonamides are antibiotics [39]. Sulfonamide antibiotics have two structural characteristics that distinguish them from nonantibiotic counterparts, namely a free amino group at N4 and a nitrogen-containing heterocyclic ring linked to N1 of the sulfonamide group (compounds B) (Figure 3). Furthermore, the discovery that sulfanilamide A has CA inhibitory properties [40] led to the discovery that corresponding derivatives C act as effective enzyme inhibitors (Figure 3) [41]. The above-mentioned structural features are essential in mediating allergic reactions to sulfonamide antibiotics [39]. A growing body of clinical evidence indicates no increased risk of reactions to nonantibiotic sulfonamides in patients with a history of allergy to sulfonamide antibiotics [39]. 

Among nonantibiotic sulfonamides, primary sulfonamides (R’-SO_2_-NH_2_) showed the most promising results due to their Zn(II) ion-binding properties; thus, they have received increased attention due to their capability to specifically inhibit CAs [42]. In fact, they form a complex in the enzyme active site with a tetrahedral geometry that is centered at the catalytic Zn (II) ion, with the N atom of the sulfonamide moiety coordinated to the bivalent metal [4,36,37,38,43]. 

**Figure 4:**
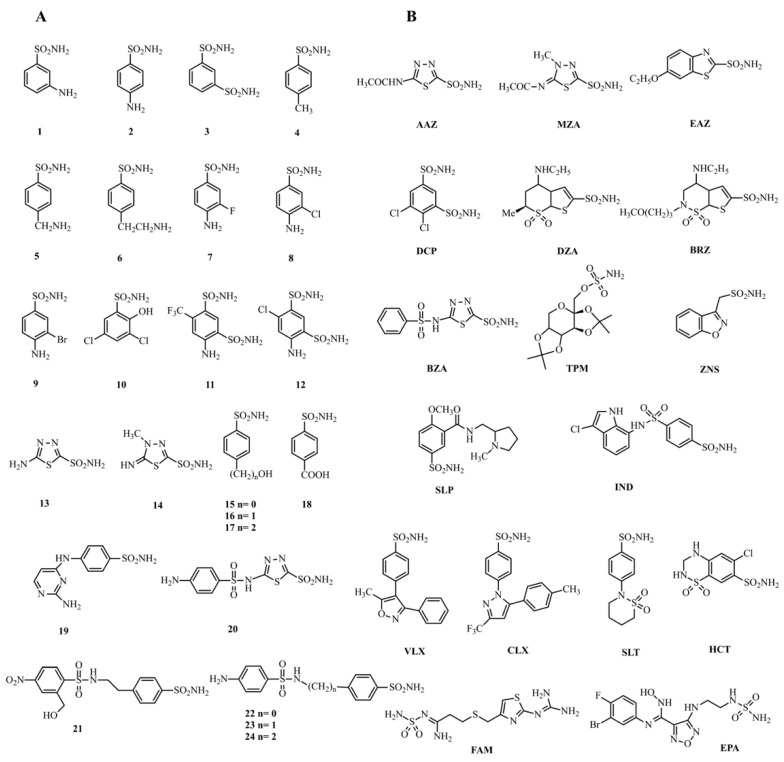
**The** structure of the compounds 1–24 (Panel **A**) and AAZ-EPA (Panel **B**) investigated as inhibitors of MpaCA.

In order to investigate the inhibition profile of MpaCA and to compare results with that of other enzyme homologues, the interaction of 41 main sulfonamides and 1 sulfamate with the enzyme from *M. pachydermatis* was investigated in vitro. The molecular structure of these compounds is shown in Figure 4. The derivatives **1–24** and **AAZ-EPA** are either simple aromatic or heterocyclic sulfonamides, and are frequently used as building blocks to create novel potent and selective pharmaceuticals [43,44]. The series AAZ-EPA (see Table 1 for their identification) involves classical CA inhibitors (CAIs) used in clinics for managing and treating glaucoma, idiopathic intracranial hypertension, altitude sickness, congestive heart failure, epilepsy, and other diseases [4,36,37,38,43,45]. 

Recently, we reported the inhibition profiles of sulfonamides against MreCA and MgCA [26,27,28,29,30,31,32,33]. Since these two fungal CAs have been proposed as new pharmacological targets for combatting fungal infection and showed a different inhibition pattern toward CAIs [26,27,28,29,30,31,32,33], we decided to explore the in vitro effect of the above-mentioned compounds on the activity of MpaCA. The data of the other two Malassezia enzymes (MreCA and MgCA) are here provided for comparison purposes. The corresponding K_I_ values are shown in Table 2. 

From the results shown in Table 2, the following conclusions can be drawn.

**Figure 5:**
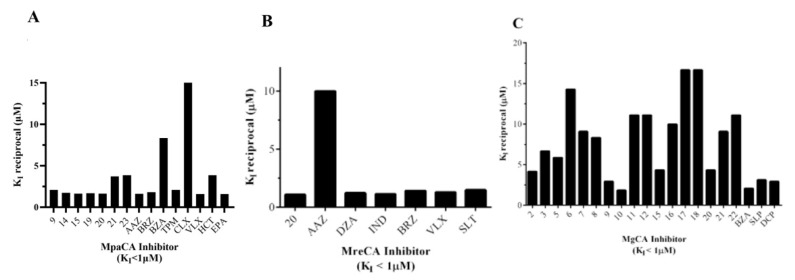
Graphical representation of the sulfonamide compounds exhibiting inhibitor activity with K_I_ values < 1.0 µM with respect to MpaCA (Panel **A**), MreCA (Panel **B**) and MgCA (Panel **C**). The K_I_ values are reported as the reciprocal to have the highest column to the best enzyme inhibitor.

1. Only fifteen drugs inhibited MpaCA with inhibition constant (K_Is_) values less than 1.0 μM. Sulfonamide inhibitors of the series 1–24, such as 9, 14, 15, 19, 20, 21, 23, and clinically used sulfonamide drugs of the series AAZ-EPA, such as AAZ, BRZ, BZA, TMP, CLX, VLX, HCT, and EPA, are significant examples. All these inhibitors showed K_I_ values in the range 0.06–0.62 µM (Table 2 and Figure 5A). MreCA showed only seven “good inhibitors” (20, AAZ, DZA, BRZ, IND, VLX, and SLT) with K_I_ values <1.0 µM (Table 2 and Figure 5B), while MgCA was well inhibited (K_I_ values 0.06–0.54 µM) by the following twenty compounds: 2, 3, 5, 6, 7, 8, 9, 10, 11, 12, 15, 16, 17, 18, 20, 21, 22, BZA, SLP and DCP (Table 2 and Figure 5C).

Interestingly, some good MpaCA inhibitors showed a moderate to limited inhibition activity on the other fungal homologous enzymes. For example, AAZ appeared as a promising inhibitor of MpaCA (K_I_ value = 0.62 µM) and MreCA (K_I_ value = 0.1 µM), but slightly inhibited MgCA (K_I_ > 10 µM) (Table 2). On the other hand, several good sulfonamide inhibitors of MgCA, with K_I_ values < 1.0 µM, showed K_I_ values in the range 1–10 µM when used against MpaCA, and were alos weak inhibitors of MreCA (Table 2), denoting how different the sulfonamide inhibition profiles were of the three fungal homologous *β*-CAs.

**Figure 6:**
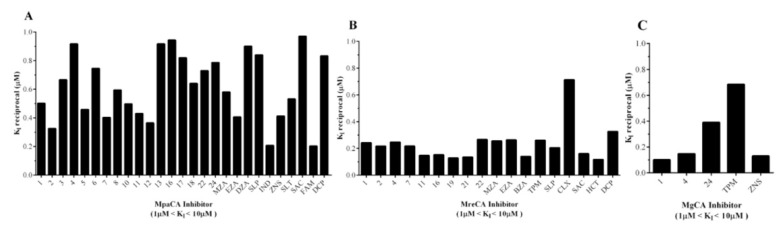
Graphical representation of the sulfonamide compounds exhibiting inhibitor activity with K_I_ values in the range between 1 and 10 µM with respect to MpaCA (Panel **A**), MreCA (Panel **B**), and MgCA (Panel **C**). The K_I_ values are reported as the reciprocal to have the highest column to the best enzyme inhibitor.

2. Many compounds of the series 1–24 and AAZ-EPA (1, 2, 3, 4, 5, 6, 7, 8, 10, 11, 12, 13, 16, 17, 18, 22, 24, MZA, EZA, DZA, SLP, IND, ZNS, SLT, SAC, FAM, and DCP) examined on MpaCA demonstrated a moderate inhibitory effect on this enzyme, with K_I_ values in the range 1.06–4.91 µM (Table 2 and Figure 6A). A number of these small molecules, namely 1, 4, 13, 24, MZA, EZA, ZNS, FAM, and SAC, were also weak inhibitors of MreCA and MgCA, showing K_I_ values higher than 1.0 µM. Figure 6B,C provide a graphical representation of these findings, showing sulfonamide inhibitors with 1 µM < K_I_s < 10 µM for these fungal enzymes.

3. As mentioned above, many of the chemicals reported in Table 2 were weak inhibitors of MreCA (Ki > 10 µM) and were already demonstrated to be effective and moderate inhibitors of the human isoenzyme II (hCA II) [26], MpaCA and MgCA, respectively. As highlighted above, MreCA showed an inhibition pattern markedly different from those of the other two homologous enzymes MpaCA and MgCA (Table 2).

## 3. Materials and Methods

### 3.1. MpaCA Production: Synthetic Gene, Cloning, Heterologous Expression, and Purification 

The synthetic *MpaCA* gene was designed in our labs and produced by Life Technologies (Invitrogen, Carlsbad, CA, USA). Briefly, the *MpaCA* gene contained NdeI and XhoI restrictions sites at the 5′- and 3′-ends, respectively; it was ligated into the expression vector pET100/D-TOPO (Invitrogen, Carlsbad, CA, USA) to form the expression vector pET100D-Topo/MpaCA, containing a nucleotide sequence encoding for a polypeptide with additional six histidines before the insertion point, for facilitating the purification of the resulting recombinant protein. To overexpress *MpaCA*, competent *E. coli* BL21 (DE3)pLysS (Agilent, Santa Clara, CA, USA) cells were transformed with pET100D-Topo/MpaCA, growing them in 1 L of LB broth at 37 °C. Isopropyl-β-D-thiogalactopyranoside (IPTG) was added to a final concentration of 1 mM, and 0.5 mM ZnSO_4_ was added after incubation for 30 min for uptake in the expressed protein. The incubation period continued for an additional 3 h at 37 °C. To verify the overexpression of *MpaCA*, the resulting bacterial suspension was tested and analyzed on 12% T SDS-PAGE, according to Laemmli [46]. At 3 h post-induction, the cellular extract was prepared by sonication at 4 °C. Following centrifugation, the supernatant containing the overrepresented MpaCA was purified using a HIS-Select HF Nickel Affinity Gel (Sigma-Aldrich, St. Louis, MO, USA), which was equilibrated with a 0.02 M phosphate buffer (pH 8.0) containing 0.01 M imidazole and 0.5 M KCl, at a flow rate of 1.0 mL/min. MpaCA was eluted from the column with 0.02 M phosphate buffer (pH 8.0) containing 0.5 M KCl and 0.3 M imidazole, at a flow rate of 1.0 mL/min [34]. The protein concentration of the obtained active fractions was determined with a Bio-Rad protein assay based on the Bradford method [47]. The enzyme resulted at least 95% pure. About 1.0 mg of final recombinant enzyme was obtained from 1 L of bacterial culture.

### 3.2. Enzyme Protonography

For protonography, SDS-PAGE was performed as described by De Luca et al. [48]. Samples were mixed in a loading buffer without 2-mercaptoethanol, and they were not boiled to avoid protein denaturation. After electrophoresis, the gel was subject to protonography to detect the hydratase activity [48].

### 3.3. Enzyme Assays

An applied photophysics stopped-flow instrument was used for assaying the CA-catalyzed CO_2_ hydration activity [49]. Phenol red (at a concentration of 0.2 mM) was used as an indicator in a buffer containing 20 mM Tris (pH 8.3), 20 mM NaClO_4_ (for maintaining a constant ionic strength), measuring the absorbance maximum of 557 nm, and following the initial rate of the CA-catalyzed CO_2_ hydration reactions for a period of 10–100 s. The CO_2_ concentrations values ranged from 1.7 to 17 mM during the determination of the kinetic parameters.

### 3.4. Inhibition Assays

At least six measurements of the original 5–10% reaction were used to assess the initial velocity for each inhibitor. The uncatalyzed rates were identically determined and detracted from the total observed rates. Stock inhibitor solutions (10–100 mM) were prepared in distilled, deionized water, and dilutions up to 0.01 mM were performed with the buffer test. Inhibitor and enzyme solutions were preincubated together for 15 min at room temperature prior to the assay, in order to allow the formation of the E–I complex or the eventual active site-mediated hydrolysis of the inhibitor. The inhibition constants, which represent the mean from at least three different determinations, were obtained by the non-linear least-squares methods using PRISM 6 and the Cheng–Prusoff equation, as reported earlier [50]. MgCA and MreCA were recombinant enzymes obtained in-house. All salts/small molecules were of the highest purity available from Sigma-Aldrich (Milan, Italy).

## 4. Conclusions

Fungal MpaCA was generated as a soluble recombinant protein using *E. coli* cells as the host. SDS-PAGE, protonography, and the stopped-flow experiments showed that MpaCA has a molecular mass of about 30 kDa and an excellent hydratase activity, converting the CO_2_ to bicarbonate and protons with a k_cat_ value of 3.8 × 10^5^ s^−1^. By using the simple aromatic/heterocyclic compounds **1–24** and the therapeutically used drugs **AAZ-EPA**, the MpaCA sulfonamide inhibition profile was determined. Among the compounds belonging to both series, only 9, 14, 15, 19, 20, 21, 23, AAZ, BRZ, BZA, TMP, CLX, VLX, HCT, and EPA inhibited MpaCA with K_I_ values < 1.0 μM, highlighting these compounds as promising compounds to be further tested for future veterinary and medical applications. The comparative analysis of the sulfonamide inhibition profiles of MpaCA, MreCA, and MgCA highlighted that MpaCA exhibits an inhibitory pattern similar to MgCA, but which is radically different from that of its homolog MreCAs. Considering the sulfonamide inhibition pattern of the human isoforms I and II (hCAI and hCA II) previously determined by our group [26], the above-mentioned fungal enzymes showed significant inhibitory differences with those of the human counterparts. 

The differences in the inhibitory effect of the sulfonamides on the three fungal enzymes can be explained considering the structural properties of each biocatalyst here studied. Sulfonamides form an enzyme–inhibitor complex with tetrahedral geometry centered at the Zn(II) ion also involving the N atom of the sulfonamide moiety. An extended network of hydrogen bonds involving amino acids of the enzyme also contributes to the inhibitor molecule anchoring to the metal ion. Besides, an interaction occurs between the aromatic/heterocycle portions of the inhibitor and the hydrophilic and hydrophobic residues present in the catalytic pocket of the enzyme [4,36,37,38,43,51]. Thus, it is reasonable to speculate that various residues present in the catalytic pocket of the different *Malassezia* enzymes may be responsible for the observed differences in the calculated K_I_ values measured for the 42 compounds described in this study. Unfortunately, none of the three fungal enzymes were crystallized and, accordingly, no structural data were available for rationalizing the enzyme’s behavior versus the investigated sulfonamides. However, these findings are encouraging because they show that, even though these CAs are very similar, there is a good chance for synthesizing inhibitors that can specifically inhibit CAs from the various fungi reported in this study as well as the human isozymes.

## Figures and Tables

**Table 1:** CAI clinically used drugs identified with their short and commercial name.

CAI	Commercial Name
AAZ	Acetazolamide
MZA	Methazolamide
EZA	Ethoxzolamide
DZA	Dorzolamide
BRZ	Brinzolamide
BZA	Benzolamide
TPM	Topiramate
SLP	Sulpiride
IND	Indisulam E7070
ZNS	Zonisamide
CLX	Celecoxib
VLX	Valdecoxib
SLT	Sulthiame
SAC	Saccharin
HCT	Hydrochlorothiazide
FAM	Famotidine
DCP	Dichlorophenamide
EPA	Epacadostat

**Table 2:** Inhibition profile of MpaCA, MreCA, and MgCA with respect to forty-one sulfonamide and one sulfamate derivatives.

	K_I_ (µM) *		
Inhibitor	MpaCA	^a^ MreCA	^a^ MgCA
1	1.99	4.12	9.8
2	3.07	4.62	0.24
3	1.5	>10	0.15
4	1.09	4.04	6.74
5	2.18	>10	0.17
6	1.34	>10	0.07
7	2.48	4.59	0.11
8	1.68	>10	0.12
9	0.48	>10	0.34
10	2.01	>10	0.54
11	2.32	6.76	0.09
12	2.74	>10	0.09
13	1.09	>10	>10
14	0.58	>10	>10
15	0.61	>10	0.23
16	1.06	6.51	0.10
17	1.22	>10	0.06
18	1.56	>10	0.06
19	0.59	7.79	>10
20	0.61	0.91	0.23
21	0.27	7.4	0.11
22	1.37	3.74	0.09
23	0.26	>10	>10
24	1.27	>10	2.56
AAZ	0.62	0.1	>10
MZA	1.72	3.9	>10
EZA	2.46	3.79	>10
DZA	1.11	0.81	>10
BRZ	0.55	0.7	>10
BZA	0.12	7.15	0.48
TPM	0.48	3.83	1.46
SLP	1.19	4.85	0.32
IND	4.82	0.87.	n.d.
ZNS	2.42	>10	7.65
CLX	0.06	1.4	>10
VLX	0.63	0.77	>10
SLT	1.88	0.67	n.d.
SAC	1.03	6.2	n.d.
HCT	0.26	8.5	n.d.
FAM	4.91	>10	n.d.
DCP	1.20	3.06	0.34
EPA	0.63	n.d.	n.d.

* Mean from three different assays as performed by stopped-flow experiments (errors were in the range of ±5–10% of the reported values). ^a^ From ref. [26]; n.d.: not detected.
